# Endothelial Dysfunction in Resuscitated Cardiac Arrest (ENDO-RCA): safety and efficacy of low-dose prostacyclin administration and blood pressure target in addition to standard therapy, as compared to standard therapy alone, in post-cardiac arrest syndrome patients: study protocol for a randomized controlled trial

**DOI:** 10.1186/s13063-016-1477-z

**Published:** 2016-08-02

**Authors:** Anna Sina P. Meyer, Sisse R. Ostrowski, Jesper Kjaergaard, Pär I. Johansson, Christian Hassager

**Affiliations:** 1 Section for Transfusion Medicine, Capital Region Blood Bank, Rigshospitalet, 2034, Blegdamsvej 9, 2100 Copenhagen, Denmark; 2 Department of Cardiology, Rigshospitalet, 2143, Blegdamsvej 9, 2100 Copenhagen, Denmark; 3 Department of Surgery, University of Texas Health Medical School, 6410 Fannin Street UPB 1100, Houston, TX 77030 USA

**Keywords:** Out-of-hospital cardiac arrest (OHCA), Endothelial dysfunction, Prostacyclin

## Abstract

**Background:**

Morbidity and mortality following initial survival of cardiac arrest remain high despite great efforts to improve resuscitation techniques and post-resuscitation care, in part due to the ischemia-reperfusion injury secondary to the restoration of the blood circulation. Patients resuscitated from cardiac arrest display evidence of endothelial injury and coagulopathy (hypocoagulability, hyperfibrinolysis), which in associated with poor outcome. Recent randomized controlled trials have revealed that treatment with infusion of prostacyclin reduces endothelial damage after major surgery and AMI. Thus, a study is pertinent to investigate if prostacyclin infusion as a therapeutic intervention reduces endothelial damage without compromising, or even improving, the hemostatic competence in resuscitated cardiac arrest patients. Post-cardiac arrest patients frequently have a need for vasopressor therapy (catecholamines) to achieve the guideline-supported blood pressure goals. To evaluate a possible catecholamine interaction with the primary endpoints of this study, included patients will be randomized into two different blood pressure goals within guideline-recommended targets.

**Methods/design:**

A randomized, placebo-controlled, double-blind investigator-initiated pilot trial in 40 out-of-hospital-cardiac-arrest (OHCA) patients will be conducted. Patients will be randomly assigned to either the active treatment group (48 hours of active study drug (iloprost, 1 ng/kg/min) or to the control group [placebo (saline) infusion]. Target mean blood pressure levels will be allocated 1:1 to 65 mmHg or approximately 75 mmHg, which gives four different permutations, namely: (i) iloprost/65 mHg, (ii) iloprost/75 mmHg, (iii) placebo/65 mmHg, and (iv) placebo/75 mmHg. All randomized patients will be treated in accordance with state-of-the art therapy including targeted temperature management.

The primary endpoint of this study is change in biomarkers indicative of endothelial activation and damage, [soluble thrombomodulin (sTM), sE-selectin, syndecan-1, soluble vascular endothelial growth factor (sVEGF), nucleosomes] and sympathoadrenal over activation (epinephrine/norepinephrine) from baseline to 48 hours post-randomization.

The secondary endpoints of this trial will include: (1) the hemostatic profile [change in functional hemostatic blood test (thrombelastography (TEG) and whole blood platelet aggregometry (multiplate)) blood cell and endothelial cell-derived microparticles]; (2) feasibility of blood pressure target intervention (target 90 %); (3) interaction of primary endpoints and blood pressure target; (4) levels of neuron-specific enolase at 48 hours post-inclusion according to blood pressure targets.

**Discussion:**

The ENDO-RCA study is a pilot study trial that investigates safety and efficacy of low-dose infusion of prostacyclin administration as compared to standard therapy in post-cardiac arrest syndrome patients.

**Trial registration:**

Trial registration at ClinicalTrials.gov (identifier NCT02685618) on 18 February 2016.

## Background

Despite efforts during the last decades to optimize cardiac arrest resuscitation techniques and post-cardiac arrest care, mortality rates are still high [1]. Survival to discharge rates from out-of-hospital cardiac arrest (OHCA) varies among studies and regions [2–4] and was reported in a recent systematic review to be 2, 6, 9 and 11 percent respectively for Asia, North America, Europe and Australia [2] with half of cardiac arrest survivors suffering from cognitive impairment [5].

The high mortality rate of patients with initial return of spontaneous circulation (ROSC) is in part due to a specific pathophysiologic process known as the *post*-*cardiac arrest syndrome* (PCAS). This term describes a global ischemia-reperfusion injury in response to whole-body ischemia following successful resuscitation [1]. On a pathophysiologic level, the ischemia-reperfusion injuries associated with PCAS involve microcirculatory dysfunction [6], vascular leakage with ensuing edema, and an increase in platelet and leukocyte adhesion to the activated/injured endothelium. The consequence of these injuries to the endothelium results in a sepsis-like inflammatory response [7–9] that ultimately determines patients’ outcome.

**Fig. 1:**
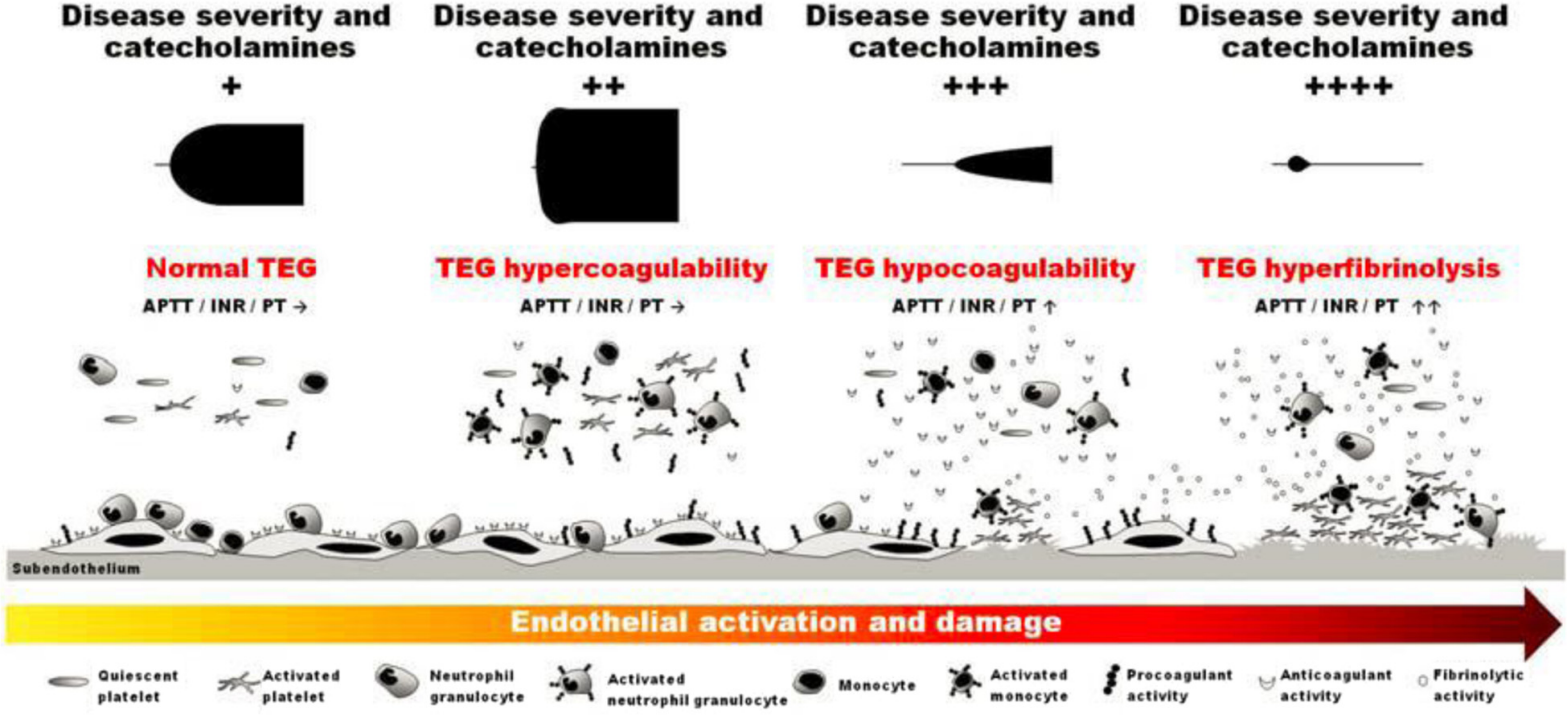
A systems biology perspective on vascular homeostasis in critical illness. Schematic illustration of the changes in the vascular system (circulating blood, vascular endothelium) with increasing disease (ischemia-reperfusion, trauma, sepsis, etc.) severity and sympathoadrenal activation (circulating catecholamines) evaluated by plasma-based (APTT, INR, PT) and functional whole blood-based (TEG) coagulation tests. Progressive endothelial activation and damage is accompanied by concurrent progressive hypocoagulability in the circulating blood. We infer (Johansson and Ostrowski) that the circulating catecholamine level dose-dependently promotes a switch from hyper- to hypocoagulability and hyperfibrinolysis in the blood to keep the progressively more procoagulant microvasculature open. From Johansson & Ostrowski [22]

Patients suffering from acute critical illness display evidence of excessive sympathoadrenal activation [10–14] and there is emerging evidence that the normal “fight-or-flight” response may become maladaptive and induce organ damage [15, 16]. Thus, increasing levels of endogenous catecholamines induce widespread dose-dependent effects on the vascular system [16]. In high concentrations catecholamines directly damage the endothelium [17, 18] and promote hypocoagulability and hyperfibrinolysis in the blood [19–21]. It is hypothesized [22] that the progressive hypocoagulability and hyperfibrinolysis observed in critically ill patients reflects an evolutionary adapted response aiming at keeping the microvasculature open in conditions with low flow, and inferred that the catecholamine surge, through its opposite directed effects on the endothelium and blood, is a critical driver of this response (Fig. 1).

This hypothesis [22] was investigated in three different types of patients characterized by acute critical illness. The outcome of these studies displayed strong and independent associations between circulating levels of catecholamines and biomarkers of endothelial activation and damage and outcome in trauma patients [23], in patients with acute myocardial infarction (AMI) [13] and in patients with severe sepsis and septic shock [24]. Several studies provide evidence of endothelial damage after successful cardiac arrest resuscitation [6, 9, 25–27] and the specific biomarkers investigated as endpoints for endothelial damage and sympathoadrenal activation in this study has recently been related to patient outcome in OHCA patients [28]. In alignment with the hypothesis described, it has been reported that patients resuscitated from cardiac arrest display evidence of coagulopathy [6, 29–31] and endothelial injury [25, 26, 32].

Current state-of-the art therapy for OHCA includes general organ support and targeted temperature management for up to 24 hours after admission in unconscious patients [33]. Therapeutic interventions directed toward the damaged endothelium may improve outcome for patients with PCAS. Prostacyclin (prostaglandin I_2_, PGI_2_) is an endogenous prostanoid which is formed and released by endothelial cells with antiplatelet, vasodilatory and cytoprotective properties [34]. Accordingly, prostacyclin is expected to be beneficial by protecting and deactivating the endothelium and by restoring vascular integrity in patients suffering from endothelial breakdown [35].

Prostacyclin is currently used for the treatment of critically ill patients with pulmonary hypertension [36] and critical limb ischemia due to its vasodilatory properties [37]. Former studies with critically ill patients that received prostacyclin [35, 38] showed results reflecting a stabilizing effect on the endothelium and hemostasis measured by endothelial biomarkers and viscoelastic hemostatic assays. These studies provide the rationale for the hypothesis that prostacyclin may be beneficial as an endothelial rescue treatment in patients suffering from endothelial breakdown such as after resuscitated cardiac arrest (RCA). From a safety point of view, the low-dose infusion of prostacyclin selected for this trial is lower than the recommended dose for approved indications. The dosage chosen for the current trial has previously been utilized without reported significant adverse side effects [38–40].

Systemic blood pressure is part of the goals in most post-cardiac arrest care protocols. Blood pressure goals have not been specifically studied and the level of evidence is generally low [41]. The recent update of the American Heart Association continues to emphasize the necessity of standardized goal-directed care but also acknowledges that existing protocols include blood pressure targets varying from 65 mmHg to as high as 80 mmHg in mean arterial pressure [42]. Studies have found a significant prevalence of impaired cerebral autoregulation post cardiac arrest, and that some patients may need significantly higher perfusion pressure to maintain cerebral blood flow post cardiac arrest [43]. The recent guideline however recommends a target blood pressure on 65 mmHg and the use of catecholamines to achieve this pressure [42]. Blood pressure and exogeneous catecholamines may consequently influence the effect of prostacyclin analogue (iloprost) on the endothelium.

### Aim

This pilot study aims to investigate (1) the relationship between the stress response and endothelial damage, (2) if prostacyclin treatment reduces endothelial damage (and organ failure) in RCA patients, (3) the effect of prostacyclin treatment on hemostasis, and (4) if blood pressure target (65 mmHg or 75 mmHg) during the intensive care unit (ICU) stay will influence the endothelial response to the study drug and levels of neuron-specific enolasis at 48 hours.

## Methods/design

### Design

This is a single-center, randomized (1:1, active:placebo), placebo-controlled, double-blind investigator-initiated pilot study in 40 out-of-hospital-cardiac-arrest (OHCA) patients investigating iloprost safety and efficacy. Furthermore, the impact of exogenous catecholamines and blood pressure targets is investigated by randomization of target blood pressures to 65 mmHg and approximately 75 mmHg. Blood pressure levels will be allocated 1:1 in a 2 × 2 factorial design, providing four different permutations: (i) iloprost/65 mHg, (ii) iloprost/75 mmHg, (iii) placebo/65 mmHg, and (iv) placebo/75 mmHg with ten patients in each group.

### Withdrawal

Participation in the ENDO-RCA trial is voluntary. Patients, relatives or scientific guardians are free to discontinue the trial at any time. The treating physician can discontinue patient participation in the trial if the physician suspects that participation affects the patient’s safety. If there is medical reason for the withdrawal, the patient should be followed medically until the condition has stabilized or terminated. The reason for withdrawal should be recorded in the patient’s case report form (CRF).

If consent for patient participation is declined by the patients’ next of kin, the patients’ general practitioner or the patient him/herself, blood samples will be destroyed according to laboratory standard operating procedure. Collected data, laboratory notes, and sample analysis results will be deleted. If consent is declined during study drug administration, the drug-infusion will be terminated. In case a patient or next of kin make a withdrawal of consent, the party will be asked for permission to use data obtained prior to withdrawal and to obtain data for the primary outcome measure. If this is achieved, the patient will be included in the final analyses. If the patient declines, all data from that patient will be destroyed. All patients who withdraw from the trial for any reason and at any time should have an end-of-trial examination. Patients will be examined for any status changes that require further follow-up. All withdrawn patients will be followed up as the remaining patients in the trial. If consent is withdrawn, the person making the withdrawal will be asked for permission to follow up for 90 days after randomization.

### Inclusion criteria

Participants in the trial must be adult patients (≥18 years of age) with OHCA of presumed cardiac cause admitted to the Department of Cardiology, 2143, Rigshospitalet, Copenhagen. Patients must be unconscious (Glasgow coma scale (GCS) <8) and have sustained ROSC (chest compressions have not been required for 20 consecutive minutes and signs of circulation persist). Temperature management is indicated.

The inclusion criteria enclose a group of critically ill patients that previously have been associated with coagulopathy and endothelial injury [6, 25, 26, 29–32] and hence, include patients with likelihood of deriving benefit from the intervention. In recent studies with patients receiving low-dose prostacyclin, the results reflects a stabilizing effect on the endothelium by lower levels of biomarkers indicative of endothelium activation and damage [35, 38]. The results suggest that patients resuscitated from cardiac arrest could benefit from low-dose infusion of prostacyclin by protecting and deactivating the endothelium and by restoring vascular integrity.

### Exclusion criteria

Patients fulfilling the following criteria will be excluded: (1) conscious patients (obeying verbal commands equal to a Glasgow Coma Score of 8 or more); (2) females of childbearing potential [unless a negative human chorionic gonadotropin (HCG) test can rule out pregnancy]; (3) patients weighing more than 135 kg; (4) in-hospital cardiac arrest (IHCA); (5) OHCA of presumed non-cardiac cause, e.g., after trauma or dissection/rupture of major artery OR cardiac arrest caused by initial hypoxia (i.e., drowning, suffocation, hanging); (6) known congenital bleeding diathesis (antithrombotic, anticoagulation medications are allowed); (7) suspected or confirmed acute intracranial bleeding; (8) suspected or confirmed acute stroke; (9) unwitnessed asystole; (10) known limitations in therapy and do-not- resuscitate order; (11) known disease-making 180-day survival unlikely; (12) known pre-arrest cerebral performance category (CPC) of 3 or 4; (13) >4 hours (240 minutes) from ROSC to screening; (14) systolic blood pressure <80 mmHg in spite of fluid loading/vasopressor and/or inotropic medication/intra-aortic balloon pump/axial flow device [If the systolic blood pressure (SBP) is recovering during the inclusion window the patient can be included.]; (15) temperature on admission <30 °C; (16) known allergy to prostacyclin analogues.

The endothelial protective effect of the study drug is believed to alleviate reperfusion injury and reduce progressive brain injury by apoptosis after OHCA. Non-comatose patients (being awake at admission or a short time to post-arrest awakening) have been related to better long-term cognitive functioning [5]. These exclusion criteria will enable exclusion of patients most unlikely to survive the intervention period of 48 hours and exclude patients with characteristics that might bias the outcomes.

### Randomization

Screening of patients to the trial will be performed by a cardiologist on duty at the Departmentof Cardiology, Rigshospitalet. A screening log will be kept at the site to determine the number of patients meeting the inclusion criteria, eligible patients, patients with consent who are randomized, and reasons why potential eligible patients were not enrolled.

Patients will only be enrolled after informed consent, but as the treatment has to be initiated earliest possible after the out-of-hospital cardiac arrest diagnosis, i.e., at a time point where patients are temporarily incompetent and the next of kin may not have reached the hospital yet and it may be outside opening hours of the general practitioner, it may be impossible to obtain surrogate consent from next of kin and general practitioner. In this situation, patients may be included after proxy consent by two independent physicians. The consent form must be signed by the participant or legally acceptable surrogate and by the investigator seeking the consent.

Randomization will be performed by a web-based site administered by a webmaster unrelated to the trial. Each patient will be assigned a unique trial and randomization number. Randomization will be generated into dynamic blocks and stratified for the clinical trial site. Patients will be randomized to receive either active treatment or placebo and target blood pressure of 65 or approximately 75 mmHg. A randomization list will be held by webmaster at the Departtment of Cardiology, which will be available to investigator after the completion of the trial. Emergency code breaking will be available and administered by the webmaster.

### Intervention

A 1:1 randomized stratification for active study drug or placebo infusion and blood pressure target for 48 hours. Patients in both randomization groups will be treated in accordance with state-of-the-art therapy including targeted temperature management. Interventions are considered emergency procedures and study drug infusion should be commenced as soon as possible after sustained ROSC, screening and randomization. Patients with active treatment will receive a low-dose prostacyclin infusion whilst the placebo group receives a dummy saline infusion for 48 hour in a total of 40 patients. The active treatment (*n* = 20 patients) will consist of continuous administration of i.v. infusions of 1.0 ng/kg/min of iloprost for 48 hours. Patients in the placebo group (*n* = 20 patients) will receive dummy saline infusion at a volume comparable to active treatment and will otherwise be treated exactly as active patients. This dosing regimen was chosen since intravenous doses of prostacyclin 0.5–2.0 ng/kg/min have been reported to be successful at achieving endothelial modulating/preserving effect with no significant hemodynamic or platelet aggregation complications [39, 40, 44]. The chosen dose of 1.0 ng/kg/min has demonstrated beneficial effects on vascular integrity in critically ill patients when administered for 3 days [40]. The 1.0 ng/kg/min dose is five- to tenfold higher than the normal endogenous production of prostacyclin from the healthy endothelium and is not expected to induce vasodilation and hypotension [45].

For investigation of the impact of blood pressure goals a supplementary randomization of target blood pressure will be made. Before the patient arrives at the ICU a randomization of using one of two identical blood pressure modules have been made, one being offset by approximately −10 mmHg. The blood pressure number displayed on the monitor will therefore be 10 mmHg lower that the actual blood pressure, thus keeping this intervention blinded to care providers, patients and relatives.

Biomarkers of endothelial damage, [soluble thrombomodulin (sTM), sE-selectin, syndecan 1, soluble vascular endothelial growth factor (sVEGF), nucleosomes] and sympathoadrenal overactivation (epinephrine/norepinephrine) together with functional hemostatic assays [thrombelastography (TEG) and whole blood platelet aggregometry (multiplate)] and blood cell- and endothelial cell-derived microparticles will be investigated together with clinical outcome measures at 6, 24, 48, 72 and 96 hours.

The study drug will be supplied by the sponsor, Pär I. Johansson MD, DMSc, MPA, Section for Transfusion Medicine, Capital Region Blood Bank, Rigshospitalet, 2034, Blegdamsvej 9, 2100 Copenhagen, Denmark.

### Blinding

To circumvent selection bias, researchers and health care personnel, will be blinded to the treatment assignment. Furthermore, to avoid investigator, health care staff and patient performance and detection bias, patients will be randomized to receive either iloprost (Ilomedin®, Bayer AG, Leverkusen, Germany) or placebo similar in color, consistency, and volume. The calibration of the identical blood pressure modules will be performed by a technician with no affiliation to the intensive care unit, thus enabling blinding of study personnel, care providers, patients and relatives. The blood pressure target intervention will be allocated according to inclusion number at initial screening and randomization of the patient. Blinded study and non-study personnel will record clinical data, analyze blood samples and perform neurocognitive assessments. All randomized patients that have received iloprost will continue to be included in the assessments of its safety and efficacy. We expect no loss to follow-up in hospital, provided that this pilot study takes place in a hospital setting where the majority of the outcomes will be evaluated. After discharge from the hospital, attrition bias will be avoided by following measures: family member contact information will be requested in case patient contact is unattainable or if the patient is incapacitated to provide information; the study coordinator will have continuous contact with the patient and family member during the ICU stay and at the time of ICU discharge to emphasize following contact (30, 90 and 180 days); the patient and family member will be notified of upcoming interviews in writing; public records will be utilized to acquire survival status on patients lost to follow-up.

### Endpoints

#### Primary outcome

The primary objective of this pilot study is safety and efficacy of low-dose iloprost administration in addition to standard therapy, as compared to standard therapy alone, in post-cardiac arrest syndrome patients. The selection of primary outcome of this study is biochemical change with markers such as change in biomarkers indicative of endothelial activation and damage (sTM, sE-selectin, syndecan-1, sVEGF, nucleosomes) and sympathoadrenal overactivation (epinephrine/norepinephrine).

#### Secondary endpoints

The secondary endpoints of this trial will include: (1) the hemostatic profile (change in functional hemostatic blood test [thrombelastography (TEG) and whole blood platelet aggregometry (multiplate) blood cell- and endothelial cell-derived microparticles]; (2) feasibility of blood pressure target intervention (target 90 %); (3) interaction of primary endpoints and blood pressure target; (4) levels of neuron-specific enolase at 48 hours post- inclusion according to blood pressure targets.

#### Tertiary endpoints

The tertiary endpoints will include: (1) days of vasopressor, ventilator, and renal replacement therapy post-randomization; (2) changes in sequential organ failure assessment (SOFA) score from baseline to 48 hours and day 4 post-randomization; (3) neurological function graded by modified Rankin Scale (mRS) and cerebral performance category (CPC) at 180 days; (4) severe bleeding [intracranial or clinical bleeding with the use of three red blood cell (RBC) units or more/24 hours]; (5) use of blood products (in ICU) post-randomization; (6) difference in day 7-, 30-, 90- and 180-day mortality between patients receiving active treatment (iloprost) and placebo; (7) estimated glomerular filtration rate (eGFR) and urine output on day 2 and 3; and (8) need for renal replacement therapy during the ICU stay.

### Assessment and registration of serious adverse reactions (SARs), serious adverse events (SAE) and suspected unexpected serious adverse reactions (SUSARs)

The serious adverse reactions predominantly reported in relation to administration of the study drug (iloprost) are allergic reactions, thrombosis, and bleeding. Occurrence of adverse events and adverse reactions will be monitored and recorded daily in the case report form (CRF).

Serious adverse reactions (SARs) and serious adverse events (SAE) will be reported immediately to the sponsor for causality evaluation and determination of possible suspected unexpected serious adverse reactions (SUSAR). The good clinical practice (GCP) unit will be given access to blinded data in the CRF to enable continuous monitoring. During the trial, the investigator will send a yearly report on the occurrence of SARs to the Danish Health and Medicines Authority and Ethics Committee.

Suspected unexpected serious adverse reactions (SUSARs) will be defined as serious adverse reactions not described in iloprost summary for product characteristics. SUSARs will be reported by the sponsor according to regulation directly to the Danish Health and Medicines Authority and Ethics Committee. SUSARs, which are fatal or life-threatening, will be reported as soon as possible and no later than 7 days after the sponsor is informed. Any other SUSARs must be reported to the Danish Health and Medicines Authority and Ethics Committee no later than 15 days from report of adverse effects.

### Statistical analysis

#### Sample size

A total 40 of patients will be recruited in a 1:1 ratio active:placebo. Blood pressure levels will be allocated 1:1 in a 2 × 2 factorial design, providing four different permutations: (i) iloprost/65 mHg, (ii) iloprost/75 mmHg, (iii) placebo/65 mmHg, and (iv) placebo/75 mmHg with ten patients in each of the four groups.

The number of patients participating is based on a power calculation. The calculation is based on data collected in a randomized study performed by Johansson et al. (manuscript in preparation), investigating the effect of prostacyclin infusion (1 ng/kg/min) on hemostasis and the endothelium in patients undergoing Whipple surgery. The primary biomarker of interest is thrombomodulin, which is used for the power calculation: the increase in circulating thrombomodulin differed significantly among the active ((mean ± SD) 0.53 ± 0.52 ng/ml) and placebo (2.14 ± 2.19 ng/ml) group post-surgery (*p* = 0.046). To detect this difference two-sided with a power of 0.85 (1-beta) and alpha of 0.05 requires *n* = 19 patients in each group, respectively (STATA IC 14.1). Twenty patients will be included in each group. All patients that receive low-dose prostacyclin infusion will be included. A total 40 patients will be recruited in a 1:1 ratio active:placebo. Patients who drop out or are withdrawn for any reason before day 4 will be replaced.

### Statistical methods

This is a pilot trial and thus all statistical tests should be considered to be exploratory. Descriptive statistics will be calculated for all endpoints. All summary statistics of continuous variables will include: n, mean with standard deviation, median with min/max and interquartile ranges. All summary statistics of frequency tables will include n, % and N, where N is the total number of patients recorded values in the corresponding group. The difference between treatment groups for continuous data will be evaluated using analysis of variance (mixed model) followed by post hoc pair-wise comparisons of means. Furthermore, delta values (numerical change in variables between time points) within and between groups will be compared by paired (Wilcoxon signed-rank test) and non-paired (Mann-Whitney *U* test) nonparametric tests. The difference between treatment groups for categorical data will be evaluated using McNemar’s test (change over time), frequency tables and chi-square statistics. The difference between treatment groups for continuous data will be evaluated using analysis of variance (mixed model) followed by post hoc pair-wise comparisons of means. Nonparametric tests, Wilcoxon rank-sum test, will be used if the assumption of normality is not fulfilled. Mortality will be evaluated applying survival statistics, i.e., Kaplan Meier plots and log-rank test and Cox proportional hazards models. Data will be tabled and summarized descriptively.

### Pilot study

ENDO-RCA is a comprehensive exploratory framework that investigates the possible beneficial role of prostacyclin on endothelial integrity in patients suffering post-cardiac arrest syndrome. Several studies in the OHCA population have been successfully conducted at the trial site (ICU at the Department of Cardiology, Rigshospitalet, Copenhagen) and the timeline for patient enrollment is anticipated to extend over 6 months. This pilot study has no planned interim analyses and the trial will not be extended. The results from ENDO-RCA, if positive, will form a basis for grant applications from the European Commission (HORIZON 2020) and the National Institutes of Health (NIH) enabling large-scale multicenter randomized clinical trials.

## Discussion

The continuously low survival-to-discharge rates after cardiac arrest necessitates further exploration of resuscitation techniques and post-cardiac arrest care. This study will attempt to address and target the harmful effects of systemic ischemia and reperfusion in patients with PCAS.

The excessive sympathoadrenal activation presented by critically ill patients [10–14] is hypothesized [22] to be a critical driver of endothelial damage [17, 18], progressive hypocoagulability and hyperfibrinolysis in the vascular system [19–21]. The stress response in critically ill patients has been linked to the development of endothelial damage and coagulopathy in three patient groups (trauma patients, AMI patients and severe sepsis and septic shock patients) of which all revealed strong and independent associations between circulating levels of catecholamines and biomarkers of endothelial activation and damage and outcome, demonstrating that patients presenting with severe endothelial dysfunction suffer from higher mortality rates as compared to patients without endothelial complications [13, 23, 24].

Prostacyclin infusion is expected to be beneficial for PCAS patients by protecting and deactivating the endothelium and by restoring vascular integrity in patients suffering from endothelial breakdown [34]. In a study of critically ill patients needing renal replacement therapy, patients receiving prostacyclin as anticoagulant in the dialysis filter had lower mortality than patients receiving heparin as anticoagulant [35]. The results were speculated to be due to a spillover effect of prostacyclin to the systemic circulation, reflecting a stabilizing effect on the endothelium. As a continuation of this finding, a randomized controlled pilot study was performed in patients undergoing percutaneous coronary intervention (PCI). The study showed that patients randomized to receive infusion of low-dose (1 ng/kg/min) prostacyclin after coronary stenting improved endothelial functionality evidenced by lower levels of the soluble endothelial biomarker sE-selectin [38]. Finally, a randomized controlled pilot study performed on patients with pancreatic cancer undergoing Whipple surgery, known to experience capillary leakage due to endothelial disintegration, patients allocated to infusion of low-dose prostacyclin presented significantly improved endothelial integrity again as evaluated by lower sE-selectin as compared to patients receiving placebo (Johansson PI. Clinical trial ET-abdominal, manuscript in preparation). This body of evidence indicates that low-dose intravenous prostacyclin may be beneficial as an endothelial rescue treatment in patients suffering from endothelial breakdown. The low-dose infusion of prostacyclin selected for this trial (1.0 ng/kg/min) is five- to tenfold higher than the normal endogenous prostacyclin production. However, the dose is lower than the recommended dose for approved indications and has been reported to be without significant adverse side effects [38–40].

Post-cardiac arrest patients most often have a need for vasopressor infusions (catecholamines) to achieve the guideline supported goal of mean arterial artery pressure of approximately 65 mmHg. Subsequently a possible interaction with the primary endpoints may exist. This will be evaluated by targeting two different goals (65 mmHg or approximately 75 mmHg) within the interval of blood pressure goals supported by the recent guidelines [42].

This study will target high-risk critically ill patients, suffering global ischemia-reperfusion injury, with likelihood to derive benefit from the immediate intervention of low-dose prostacyclin infusion. The included patient group provides a pertinent background to investigate circulating catecholamines, endothelial damage and coagulopathy in low/ceased blood flow and the protective and stabilizing effects of prostacyclin on the endothelium.

### Trial status

Inclusion of patients is scheduled to commence February 2016 and expected to be completed by September 2016. This research project is a multidisciplinary collaboration between laboratory and clinical medical expertise encompassing a prospective, randomized, double-blinded pilot study in PCAS patients, and will be conducted with established partners in the Blood Bank and in the ICU at the Department of Cardiology, Rigshospitalet, Copenhagen.

## Abbreviations

AE, adverse event; AMI, acute myocardial infarction; CPC, cerebral performance category; eGFR, estimated glomerular filtration rate; GCP, good clinical practice; GCS, Glasgow coma scale; ICU, intensive care unit; iloprost, prostacyclin analogue; mRS, modified Rankin Scale; Multiplate, whole blood platelet aggregometry; OHCA, out-of-hospital cardiac arrest; PCAS, post-cardiac arrest syndrome; PCI, percutaneous coronary intervention; PGI2, prostacyclin; RBC, red blood cells; ROSC, return of spontaneous circulation; SAE, serious adverse event; SOFA, sequential organ failure assessment; sTM, soluble thrombomodulin; SUSAR, suspected unexpected serious adverse reaction; sVEGF, soluble vascular endothelial growth factor; TEG, thrombelastography
